# Trends in Modifiable Risk Factors Contributing to Ischemic Heart Disease Mortality in Afghanistan: Estimates From the Global Burden of Disease Study 2023 (1999-2023)

**DOI:** 10.7759/cureus.117727

**Published:** 2026-10-10

**Authors:** Ahmad Khan, Melanie Tidman

**Affiliations:** 1 Faculty of Medicine, Paktia University, Gardez, AFG; 2 Health Sciences, A.T. Still University, Albuquerque, USA

**Keywords:** afghanistan, global burden of disease, hypertension, iran, ischemic heart disease, modifiable risk factors, mortality trends, multiple regression, pakistan, tajikistan

## Abstract

Background: Ischemic heart disease (IHD) is the leading cause of death, and the majority of this burden can be attributed to modifiable risk factors. Although surveys show that these risk factors are prevalent in Afghanistan, no study has used Global Burden of Disease (GBD) methodology to describe their contribution to IHD mortality. The study examined trends (1999-2023) in 14 selected behavioral, dietary, and metabolic risk factors for IHD mortality using the GBD 2023 estimates.

Methodology: This retrospective analysis used GBD 2023 estimates of deaths from IHD in Afghanistan attributable to 14 selected behavioral, dietary, and metabolic risk factors and to the GBD "all risk factors" composite, which also includes exposures outside these 14 (e.g., air pollution), covering 1999-2023 (both sexes; all ages and age-standardized). The number (N) and percentage (%) attributable to each risk factor are reported. Trends in age-standardized death rates were examined with a segmented log-linear trend analysis written by the authors as a permutation-based approximation to the NCI Joinpoint approach; the NCI software was not used. The average annual percentage change (AAPC) and its 95% confidence interval (CI) are conditional on the selected model. A multiple linear regression was used only as a descriptive comparison of average rates by risk factor category, sex, and calendar year. A p-value of 0.05 or less was considered significant, with no adjustment for multiple comparisons, so the trend results are exploratory.

Results: From 1999 to 2023, the age-standardized IHD death rate for all GBD risk factors combined (the GBD composite) went down from 275.94 to 230.49 per 100,000. This is a drop of 16.5% (AAPC: −0.77%, 95% CI: −0.90 to −0.63; p < 0.001). We found at least one joinpoint in 41 of the 45 series, and most of them were around 2009 and 2013. High systolic blood pressure (11,969.1 deaths, or 55.17% of all IHD deaths in 2023) and high low-density lipoprotein (LDL) cholesterol (5,875.8 deaths, or 27.12%) remained the leading factors among the 14, both of which were decreasing (AAPC: −0.95% and −1.14%; both p < 0.001). The rates associated with a diet high in sugar-sweetened beverages (AAPC: +1.28%, 95% CI: +0.94 to +1.63; p < 0.001) and with smoking (AAPC: +0.37%; p < 0.001) increased, whereas the increases linked to a diet high in processed meat, sodium, and secondhand smoke were not significant in the segmented model (all p > 0.05). The age-standardized rates were higher among females, while the smoking rates were higher among males (25.83 as compared with 5.09 per 100,000). In the descriptive regression model (N = 700; R² = 0.963), average rates differed by risk factor category, calendar year, and sex (all p < 0.05), with high systolic blood pressure having the largest coefficient (B = +140.92; p < 0.001).

Conclusions: In Afghanistan, metabolic risk factors, especially high systolic blood pressure and high LDL cholesterol, were the largest contributors to IHD deaths among the 14 selected risk factors. The composite age-standardized rate declined, with a common inflection around 2009-2013 that may reflect GBD modeling inputs. The rates attributable to sugar-sweetened beverages and to smoking increased, which points to an emerging threat that should be the subject of targeted prevention measures, with ongoing efforts to control hypertension and lipid levels. These exploratory trend estimates need confirmation with the NCI Joinpoint software.

## Introduction

Ischemic heart disease (IHD) is one of the main causes of death around the world, and in the majority of instances, this burden is the result of a few modifiable risk factors rather than of the non-modifiable ones, such as age or genetic predisposition [1-3]. The 2023 report of the Global Burden of Disease (GBD) study included estimates of mortality by cause for 292 different causes in 204 countries and territories from 1990 through to 2023. The accompanying GBD 2023 risk factors analysis provided a comparative risk assessment for 88 risk factors, enabling the number and proportion of deaths from a given cause in a particular country and year to be estimated for each risk factor [1,4].

A recent global analysis based on GBD 2021 data showed that high systolic blood pressure and high levels of low-density lipoprotein (LDL) cholesterol were the two main risk factors responsible for IHD deaths worldwide, accounting for 52.2% and 30.1% of global IHD deaths, respectively. Between 1990 and 2021, the global age-standardized death rate from IHD decreased by approximately 31.6%, even though the number of cases continued to increase due to population growth and aging [2]. This method has also been used at a subnational level, for example, to examine IHD deaths in US states [5].

For each of these main exposures, there is a well-documented, direct biological link with cardiovascular risk: there is a graded, continuous association between systolic blood pressure and cardiovascular mortality as found in large modern cohort studies [6], and lowering LDL cholesterol reduces cardiovascular events in a dose-dependent, causal manner as demonstrated by genetic, epidemiological, and randomized-trial evidence [7].

Data from Afghanistan's national STEPwise approach to noncommunicable disease risk factor surveillance (STEPS)-based survey in 2018 showed that one quarter of adult Afghans aged 18-69 had high blood pressure, 8% had high blood glucose, 42.8% were overweight or obese, and 8.6% were current smokers of tobacco [8]. A systematic review and meta-analysis of the non-communicable disease risk factors in Afghanistan also indicated a high under-diagnosed level of hypertension, dysglycemia, and excess adiposity among adults [9].

Reports after the 2021 political change describe further decreases in donor funding, loss of health workers, and restricted access to routine and long-term disease care in a health system already strained by 40 years of instability, all of which could affect control of cardiometabolic risk factors [10,11]. However, to our knowledge, no study has used the GBD comparative risk assessment method to describe the year-by-year trend in IHD mortality attributable to modifiable risk factors in Afghanistan, nor has it assessed how the relative contribution of each risk factor has changed by sex over this multi-decade period.

Afghanistan's burden has also not been set against that of its neighbors. Pakistan, Iran, and Tajikistan share borders, diets, and cultural features with Afghanistan but differ widely in health-system capacity and in the completeness of the death-registration data behind their GBD estimates [12], so they are useful for judging whether Afghanistan's trends are unusual or regional.

Out of the 88 risk factors evaluated in the broader GBD comparative risk assessment, we selected 14 modifiable behavioral, dietary, and metabolic risk factors with estimates of attributable IHD burden for Afghanistan. These are smoking, exposure to secondhand smoke, high fasting plasma glucose, high systolic blood pressure, a high body-mass index, a diet low in fruits, a diet low in vegetables, a diet low in whole grains, a diet high in processed meat, a diet high in sugar-sweetened beverages, a diet high in sodium, low physical activity, kidney dysfunction, and high LDL cholesterol. Each risk factor was assessed in a separate category, along with the GBD composite "all risk factors" category, which gives the joint burden of all risk factors that GBD links to IHD, including exposures that are not among the 14 selected here (for example, air pollution). The composite is therefore broader than the 14 risk factors, and we keep the two apart throughout this paper.

To put the 14 risk factors, the two sexes and the calendar years on one scale, we also fitted a multiple linear regression. It is used here as a descriptive comparison of average rate levels. It is not a model of the time trend of each risk factor.

The study had four aims to (1) describe the trend in the age-standardized rates of IHD deaths due to 14 selected behavioral, dietary, and metabolic risk factors in Afghanistan between 1999 and 2023; (2) compare the burden and trend of these risk factors according to sex during the study period; (3) compare, in a descriptive multiple linear regression, the average age-standardized rate of IHD deaths across risk factor category, sex, and calendar year; and (4) compare the GBD composite and leading risk-factor trends in Afghanistan with those in Pakistan, Iran, and Tajikistan.

## Materials and methods

Study design, setting, and data source

This secondary descriptive analysis uses aggregate, de-identified data from the GBD Study 2023, which was accessed via the GBD Results Tool [13]. The data on deaths from IHD due to the 14 selected risk factors and the GBD composite in Afghanistan pertain to the calendar years from 1999 to 2023 inclusive (thus representing 25 annual data points). The GBD estimates are obtained through modeling and comprise only aggregate, population-level statistics; therefore, institutional ethical review board approval was not required.

Inclusion and exclusion criteria

The GBD Results Tool returns only the categories selected in its menus, so the exclusions below were made at the download stage rather than by screening the extracted records. The query specified deaths as the measure; IHD as the cause; Afghanistan as the location; the 14 risk factors listed under Variables together with "all risk factors"; number, percent, and rate as the metrics; all ages and age-standardized as the age groups; male, female, and both sexes; and the years 1999-2023. Deaths were the only measure selected. The study period was set at 1999-2023, giving 25 annual data points; earlier GBD years were not selected. Of the GBD risk factors linked to IHD, the environmental and occupational exposures (ambient and household air pollution, lead exposure and non-optimal temperature) were not selected. These exposures can also be changed, mainly through policy and population-level action, and we do not regard them as non-modifiable. They were left out only because this study was designed to focus on behavioral, dietary, and metabolic risk factors. Their burden is still counted inside the composite "all risk factors" category, which was retained because GBD reports it as a single jointly modeled estimate. The only record-level exclusion was the removal of 100 duplicate rows for Afghanistan in 2009 that the download of the regional file produced. No record was excluded on the basis of its uncertainty interval, and all 5,625 Afghanistan rows returned by the query were analyzed.

Variables

Each record in the dataset carried the following fields: population group (all population); measure (deaths); metric; risk factor; cause (IHD); location (Afghanistan); age group (all ages or age-standardized); sex (male, female, both); calendar year; the point estimate (Value); and the 95% uncertainty interval bounds (upper, lower) from the GBD 2023 models. Three metrics were available: the number of deaths, the percent of IHD deaths attributable to the risk factor, and the rate per 100,000. Rates are crude for the all-ages stratum and age-standardized by the direct method [14], using the GBD standard population, for the age-standardized stratum.

The analysis covered 14 individual behavioral, dietary, and metabolic risk factors: smoking, secondhand smoke, high fasting plasma glucose, high systolic blood pressure, high body-mass index, diet low in fruits, diet low in vegetables, diet low in whole grains, diet high in processed meat, diet high in sugar-sweetened beverages, diet high in sodium, low physical activity, kidney dysfunction, and high LDL cholesterol, together with the GBD composite "all risk factors" category, which is the jointly modeled burden of all GBD risk factors linked to IHD (the 14 analyzed here and the remaining GBD risk factors, such as air pollution). The composite is therefore not a summary of the 14 risk factors alone. Each of these 14 exposures has prior epidemiologic or clinical-trial evidence linking it independently to cardiovascular risk, including secondhand smoke exposure [15], low fruit and vegetable intake [16], low whole-grain intake [17], high processed/red meat intake [18], high dietary sodium intake [19], and low physical activity [20].

Data cleaning and validation

The file had 5,625 rows. This is the number of rows we expected for the query (15 risk categories, 3 sexes, 25 years, and 5 combinations of metric and age group; there are three metrics for all ages but only two for age-standardized, as GBD gives no age-standardized counts). No row was duplicated when we compared rows on metric, risk factor, age group, sex, and year. All point estimates were within their 95% uncertainty intervals. No field used in the analysis had a missing value.

Statistical analysis

The analyses were carried out using Python 3.11.15 (together with pandas 3.0.2, NumPy 2.4.4, and SciPy 1.17.1; Python Software Foundation, Wilmington, DE); the figures were produced with Matplotlib 3.10.9. The regression, variance inflation factors, and Durbin-Watson statistic were computed directly with NumPy and SciPy. No proprietary software was employed, and fixed random seeds were used so that the permutation results are repeatable. We downloaded the data from the GBD Results Tool in May 2026. We used a fixed random seed (2023) for the permutation tests. The corresponding author can provide the Python code and data file upon request.

The numbers of IHD deaths due to each risk factor in 1999 and 2023 (for both sexes and for all ages) are given as N (%), and the percentage change is calculated using the formula

*((N₂₀₂₃ &minus; N₁₉₉₉)/N₁₉₉₉) &times; 100 *.

Trend Analysis

For each risk factor and sex, the annual percent change in the age-standardized IHD death rate (per 100,000) from 1999 to 2023 was estimated by a segmented log-linear trend analysis (piecewise-linear regression on the natural logarithm of the rate). We wrote this analysis ourselves in Python as a permutation-based approximation to the approach of the National Cancer Institute (NCI) Joinpoint Regression Program [21]. The NCI software was not used, and our procedure should not be taken as equivalent to it. The model used piecewise-linear ordinary least squares with up to two joinpoints, at least four observations in each end segment and at least four years between joinpoints; joinpoint positions were found by a grid search that minimized the residual sum of squares. The number of joinpoints was chosen by a permutation test (4,499 permutations, fixed seed), and a joinpoint was kept only if its p-value was below 0.05 [22]. The test was applied in two steps (0 versus the best of one or two joinpoints, then one versus two), each at a nominal 0.05 level, without the overall correction for the search that the NCI program applies. The average annual percent change (AAPC) is the segment-length-weighted average of the log-slopes, calculated as

$AAPC = (e^(\Sigmawᵢ&beta;ᵢ) &minus; 1) &times; 100$

together with its 95% confidence interval and p-value obtained from the delta method; in the case where no joinpoint is selected, a single log-linear trend is obtained. The confidence intervals and p-values of the AAPC are conditional on the selected model. They treat the number and position of the joinpoints as fixed and do not include the uncertainty that comes from choosing them from the data, so they are probably too narrow. For this reason, we report the joinpoints and the AAPCs as exploratory estimates. The AAPC was tested against zero with a t-test on the log scale (t = AAPC estimate ÷ delta-method standard error, with n − k − 2 degrees of freedom, where k is the number of joinpoints); the permutation-test p-values for the number of joinpoints are given in the tables. For comparison, a single log-linear trend without joinpoints was also fitted to every series.

The age-standardized rate of IHD was regressed on risk factor category, sex and calendar year using ordinary least squares [23]. The data for males and females were combined, and the "both sexes" and "all risk factors" categories were omitted, resulting in 700 observations (14 risk factors × 25 years × 2 sexes). The model used was Rate = β₀ + β₁(Year) + Σβᵢ(Risk factor) + βₛ(Sex = Female) + ε, with smoking and male serving as the reference categories. This model gives one common linear yearly change for all 14 risk factors and both sexes. We did not add year-by-risk-factor or year-by-sex interaction terms, because the trend of each risk factor in each sex is already estimated separately in the segmented analysis. The regression is therefore only a descriptive comparison of average rate levels. Its year coefficient is an average over risk factors that move in different directions, and it should not be read as the time pattern of any single risk factor. Calendar year was centered at its mean (2011), meaning that the intercept corresponds to the average year.

Significance was assessed by means of two-sided t-tests; the model's fit was examined using R², adjusted R², and the F-test. Multicollinearity was investigated using the variance inflation factor (VIF), and autocorrelation was checked with the Durbin-Watson statistic [24]. Since each group provides multiple annual measurements, a Durbin-Watson value much below 2 was expected; the statistic is given only for diagnosis, and the standard errors should be interpreted as indicating the strength of the association rather than as fully design-adjusted inferences.

For a regional comparison, the same GBD 2023 data were extracted (IHD deaths attributable to the 14 risk factors and the composite category; for males, females, and both sexes; for all ages and age-standardized; covering the period 1999-2023) for Pakistan, Iran (Islamic Republic of), and Tajikistan. The combined dataset contained 22,600 rows; 100 rows (corresponding to Afghanistan in 2009) were exact duplicates resulting from the download and were therefore deleted, so that 22,500 rows remained, a number that agreed with the expected cross-tabulation (4 countries × 15 risk categories × 3 sexes × 25 years × 5 metric-by-age combinations) and with no point estimate lying outside its uncertainty interval.

The same segmented trend procedure was used for the both-sex, age-standardized rates relating to the composite category and to seven individual risk factors: high systolic blood pressure, high LDL cholesterol, high fasting plasma glucose, kidney dysfunction, high body-mass index, a diet high in sugar-sweetened beverages and smoking. A table also presents the rates in 1999 and 2023, the relative change, the 2023 attributable percentage, the joinpoint years, and the AAPC. A figure displays the composite trajectories. The sex-stratified and regression analyses were not repeated for the comparator countries. These countries served as a benchmark rather than as the subjects of the study.

We took a two-sided p ≤ 0.05 as significant and marked it with an asterisk (*) in all tables and figures; p-values below 0.001 appear as "< 0.001*". Many tests were carried out (45 risk factor-by-sex series for Afghanistan, 24 series for the comparator countries, the permutation tests for the joinpoints, and the regression coefficients), and the p-values were not adjusted for multiple comparisons. Some significant results may therefore be false positives, and the individual trend tests are exploratory. As a simple check, we compared the 45 Afghanistan AAPC p-values with a Bonferroni threshold of 0.05/45 = 0.0011. Rates are per 100,000 population and age-standardized unless we say otherwise. The cause-specific burden is given as N (%).

## Results

Table 1 gives the population and the total IHD deaths by sex in 1999 and 2023.

**Table 1:** Population and ischemic heart disease deaths in Afghanistan (1999 and 2023) by sex (GBD 2023 estimates). Population is the denominator implied by the GBD 2023 estimates (attributable deaths divided by the crude attributable rate). Population is given in millions; deaths are given as absolute numbers (not thousands); rates are per 100,000 population. Total IHD deaths were derived from the attributable deaths and the attributable percentage. Crude rate = total IHD deaths per 100,000 population. Age-standardized rates use the GBD standard population. "All risk factors" is the GBD composite. It includes the 14 selected risk factors and other GBD risk factors that were not analyzed one by one (for example, air pollution). IHD: ischemic heart disease; GBD: Global Burden of Disease Table developed by the authors from GBD 2023 data obtained through the GBD Results Tool [13].

Characteristic	1999 Males	1999 Females	1999 Both Sexes	2023 Males	2023 Females	2023 Both Sexes
Population (millions), implied by GBD 2023	8.96	8.77	17.73	18.46	17.78	36.24
Total IHD deaths, all ages	8,858.8	9,849.8	18,711.2	10,464.7	11,248.8	21,715.2
Crude IHD death rate per 100,000	98.85	112.28	105.51	56.68	63.26	59.92
IHD deaths attributable to all risk factors, N (%)	8,341.5 (94.16)	9,417.4 (95.61)	17,758.8 (94.91)	9,773.0 (93.39)	10,613.2 (94.35)	20,386.2 (93.88)
Age-standardized attributable IHD death rate per 100,000	246.40	313.80	275.94	217.95	243.86	230.49

From 1999 to 2023, the number of deaths from IHD in Afghanistan (for both sexes and all age groups) attributable to all GBD risk factors combined (the GBD composite, which is broader than the 14 selected risk factors) increased from 17,758.8 (94.91% of all IHD deaths) to 20,386.2 (93.88%), which is a rise of 14.8%. Among the 14 selected risk factors, high systolic blood pressure was the main contributor throughout this period, responsible for 11,969.1 deaths (55.17%) in 2023, and then high LDL cholesterol (5,875.8 deaths, 27.12%), followed by kidney dysfunction (3,329.9 deaths, 15.32%), high fasting plasma glucose (3,183.3 deaths, 14.67%), and high body-mass index (2,797.1 deaths, 12.85%). The greatest relative increases in the number of attributable deaths were seen for a diet high in sugar-sweetened beverages (+139.1%), smoking (+80.9%), diet high in processed meat (+49.4%), diet low in whole grains (+47.0%), and high body-mass index (+46.6%). Table 2 provides the complete comparison for all 14 risk factors.

**Table 2:** Number and percentage of ischemic heart disease deaths attributable to the 14 selected risk factors and to the GBD composite of all risk factors, Afghanistan (1999 vs. 2023, both sexes, all ages). Data are shown as N (%). N is the estimated number of ischemic heart disease (IHD) deaths attributed to each risk factor, for both sexes and all ages. The deaths are whole counts, not thousands. % is the share of all IHD deaths in Afghanistan attributed to that risk factor in that year. % change in N is the change in the death count from 1999 to 2023. The percentages use total IHD deaths as the denominator; the population at risk was 17.7 million in 1999 and 36.2 million in 2023 (Global Burden of Disease (GBD) 2023 estimates). "All risk factors" is the joint estimate for all GBD risk factors linked to IHD, including some that are not among the 14 rows above (for example, air pollution). It is not the sum of the rows above it, because the risk factors overlap and act through one another. Table made by the authors from GBD 2023 data taken from the GBD Results Tool [13].

Risk Factor	1999, N (%)	2023, N (%)	% Change in N, 1999-2023
Total IHD deaths (denominator)	18,711.2 (100%)	21,715.2 (100%)	+16.1%
High systolic blood pressure	11,132.1 (59.40%)	11,969.1 (55.17%)	+7.5%
High LDL cholesterol	5,168.3 (27.69%)	5,875.8 (27.12%)	+13.7%
Kidney dysfunction	2,848.2 (15.20%)	3,329.9 (15.32%)	+16.9%
High fasting plasma glucose	2,516.8 (13.47%)	3,183.3 (14.67%)	+26.5%
High body-mass index	1,908.3 (10.18%)	2,797.1 (12.85%)	+46.6%
Diet low in whole grains	1,392.8 (7.42%)	2,046.7 (9.40%)	+47.0%
Diet low in fruits	1,886.7 (10.09%)	1,998.1 (9.14%)	+5.9%
Smoking	1,012.7 (5.43%)	1,832.2 (8.44%)	+80.9%
Diet low in vegetables	1,454.1 (7.76%)	1,637.1 (7.52%)	+12.6%
Secondhand smoke	955.7 (5.13%)	1,330.5 (6.14%)	+39.2%
Low physical activity	570.2 (3.06%)	680.4 (3.13%)	+19.3%
Diet high in sodium	441.1 (2.34%)	575.9 (2.66%)	+30.6%
Diet high in processed meat	35.4 (0.19%)	52.9 (0.24%)	+49.4%
Diet high in sugar-sweetened beverages	6.4 (0.03%)	15.3 (0.07%)	+139.1%
All risk factors (GBD composite)	17,758.8 (94.91%)	20,386.2 (93.88%)	+14.8%

The total of the 14 separate risk factors sums to well over 100% (167.4% in 1999 and 171.9% in 2023) since each factor is calculated on its own. Many of the risk factors act through shared pathways; a high body-mass index, for example, raises IHD risk partly through blood pressure, LDL cholesterol, and fasting glucose. Only the "All risk factors" figure is the jointly modeled, non-overlapping estimate, and it also covers GBD risk factors outside the 14: 17,758.8 deaths (94.91%) in 1999 and 20,386.2 (93.88%) in 2023. The number of deaths not attributable to any GBD risk factor, 952.4 (5.09%) in 1999 and 1,329.0 (6.12%) in 2023, is due to non-modifiable factors or to risk factors that GBD does not quantify.

Although the number of deaths rose, the age-standardized rate fell. The combined ("All risk factors") age-standardized IHD death rate for both sexes fell from 275.94 to 230.49 per 100,000 between 1999 and 2023, a decrease of 16.5% (segmented-model AAPC: −0.77%, 95% CI: −0.90 to −0.63; p < 0.001; see Methods and Table 3). A rising number of deaths together with a falling age-standardized rate is consistent with population growth and aging. We did not carry out a formal decomposition, so we cannot say how much of the change in deaths comes from population growth, from aging, or from changes in the underlying age-specific rates. Afghanistan's population grew from about 19.9 million in 1999 to 41.5 million in 2023 [25]. The GBD population estimates behind the rates are lower (17.7 and 36.2 million; Table 1), but they show the same doubling. The higher number of deaths, taken alone, does not mean the risk-factor profile has worsened. Figure 1 shows the divergence and differing trends exhibited by the individual risk factors over the full 25-year period.

**Table 3:** Sex-stratified age-standardized ischemic heart disease death rate in 1999 and 2023, with exploratory average annual percent change (AAPC) from the segmented log-linear trend analysis, by risk factor (Afghanistan). The segmented analysis is the authors' own permutation-based approximation to the National Cancer Institute (NCI) Joinpoint approach and is not the output of the NCI software. Rates are age-standardized IHD deaths per 100,000 population attributable to each risk factor, taken from the GBD 2023 point estimates. AAPC is the average annual percent change for 1999-2023, a weighted average of the slopes of the joinpoint segments, each weighted by its length in years; CI is the 95% confidence interval from the delta method. t (df): t statistic and degrees of freedom for the test that the AAPC differs from zero (t-test on the log-linear slope, delta-method standard error). Joinpoint(s): calendar year(s) at which a statistically significant change in trend was selected by permutation test (maximum two joinpoints; 4,499 permutations); values in parentheses after the joinpoint years are the permutation-test p-values for 0 versus ≥1 joinpoint and, where applicable, 1 versus 2 joinpoints; a joinpoint was retained when p < 0.05; "None (linear trend)" indicates that a single log-linear segment was retained. The CI and p-value of each AAPC are conditional on the selected number and position of joinpoints and do not include model-selection uncertainty. P-values are not adjusted for multiple comparisons. "All risk factors" is the jointly modeled GBD composite estimate, which includes GBD risk factors outside the 14, and is not the sum of the individual risk factors. LDL: low-density lipoprotein, * p ≤ 0.05 Table developed by the authors from GBD 2023 data obtained through the GBD Results Tool [13].

Risk Factor	1999 Rate	2023 Rate	AAPC % (95% CI)	t (df)	p-value	Joinpoint(s) (Permutation p)
BOTH SEXES						
High systolic blood pressure	174.93	140.15	-0.95 (-1.09 to -0.81)	-14.05 (21)	<0.001*	2009, 2013 (0.007/0.010)
High LDL cholesterol	74.11	55.99	-1.14 (-1.30 to -0.98)	-14.82 (23)	<0.001*	None (linear trend) (0.120)
Kidney dysfunction	46.80	41.77	-0.45 (-0.57 to -0.33)	-7.78 (21)	<0.001*	2009, 2013 (0.001/<0.001)
High fasting plasma glucose	40.44	37.94	-0.26 (-0.39 to -0.13)	-4.18 (21)	<0.001*	2009, 2013 (<0.001/0.002)
High body-mass index	25.42	25.58	-0.03 (-0.19 to +0.13)	-0.37 (21)	0.715	2009, 2013 (0.001/0.011)
Diet low in whole grains	20.01	19.76	-0.10 (-0.24 to +0.04)	-1.43 (22)	0.166	2015 (0.002/0.104)
Diet low in fruits	27.21	18.74	-1.55 (-1.73 to -1.38)	-18.06 (22)	<0.001*	2020 (0.028/0.664)
Diet low in vegetables	21.63	16.54	-1.21 (-1.35 to -1.08)	-18.33 (22)	<0.001*	2009 (0.007/0.233)
Smoking	13.53	14.78	+0.37 (+0.22 to +0.53)	4.96 (22)	<0.001*	2014 (0.002/0.140)
Secondhand smoke	14.63	14.76	+0.04 (-0.10 to +0.17)	0.55 (21)	0.586	2010, 2014 (0.003/0.003)
Diet high in sodium	9.04	8.82	-0.05 (-0.19 to +0.08)	-0.84 (21)	0.411	2009, 2013 (<0.001/<0.001)
Low physical activity	9.53	8.58	-0.43 (-0.55 to -0.30)	-7.03 (21)	<0.001*	2009, 2013 (<0.001/<0.001)
Diet high in processed meat	0.49	0.46	-0.07 (-0.23 to +0.08)	-0.98 (21)	0.339	2009, 2014 (<0.001/<0.001)
Diet high in sugar-sweetened beverages	0.08	0.11	+1.28 (+0.94 to +1.63)	7.71 (21)	<0.001*	2002, 2015 (<0.001/<0.001)
All risk factors	275.94	230.49	-0.77 (-0.90 to -0.63)	-11.95 (21)	<0.001*	2009, 2013 (0.003/0.003)
MALES						
High systolic blood pressure	146.61	130.22	-0.54 (-0.68 to -0.39)	-7.76 (21)	<0.001*	2009, 2013 (0.005/0.026)
High LDL cholesterol	59.69	48.28	-0.96 (-1.12 to -0.80)	-12.39 (23)	<0.001*	None (linear trend) (0.115)
Kidney dysfunction	40.35	37.78	-0.30 (-0.43 to -0.17)	-4.68 (21)	<0.001*	2010, 2014 (0.002/0.014)
High fasting plasma glucose	35.95	36.40	+0.01 (-0.13 to +0.14)	0.10 (21)	0.925	2009, 2013 (0.002/0.010)
High body-mass index	18.16	21.74	+0.74 (+0.58 to +0.89)	9.83 (22)	<0.001*	2008 (0.002/0.095)
Diet low in whole grains	17.18	18.09	+0.11 (-0.04 to +0.27)	1.53 (22)	0.141	2014 (0.030/0.345)
Diet low in fruits	26.81	20.06	-1.43 (-1.60 to -1.25)	-16.34 (23)	<0.001*	None (linear trend) (0.064)
Diet low in vegetables	19.66	15.73	-1.09 (-1.24 to -0.94)	-15.23 (22)	<0.001*	2010 (0.003/0.157)
Smoking	22.34	25.83	+0.54 (+0.39 to +0.70)	7.30 (22)	<0.001*	2014 (<0.001/0.078)
Secondhand smoke	15.00	13.77	-0.41 (-0.55 to -0.26)	-5.91 (21)	<0.001*	2010, 2014 (0.008/0.012)
Diet high in sodium	10.58	10.67	+0.04 (-0.11 to +0.19)	0.62 (21)	0.542	2009, 2013 (<0.001/<0.001)
Low physical activity	4.88	4.77	-0.10 (-0.23 to +0.02)	-1.68 (21)	0.107	2009, 2013 (<0.001/0.005)
Diet high in processed meat	0.48	0.48	+0.13 (-0.02 to +0.28)	1.81 (21)	0.084	2009, 2014 (<0.001/<0.001)
Diet high in sugar-sweetened beverages	0.09	0.13	+1.54 (+1.20 to +1.87)	9.70 (21)	<0.001*	2002, 2015 (<0.001/<0.001)
All risk factors	246.40	217.95	-0.57 (-0.71 to -0.42)	-8.19 (21)	<0.001*	2010, 2014 (0.009/0.025)
FEMALE						
High systolic blood pressure	209.89	151.37	-1.38 (-1.52 to -1.24)	-20.17 (21)	<0.001*	2009, 2013 (0.010/0.009)
High LDL cholesterol	91.70	63.46	-1.43 (-1.60 to -1.27)	-17.93 (23)	<0.001*	None (linear trend) (0.082)
Kidney dysfunction	54.74	45.78	-0.70 (-0.82 to -0.58)	-12.19 (21)	<0.001*	2009, 2014 (<0.001/<0.001)
High fasting plasma glucose	45.99	39.47	-0.60 (-0.73 to -0.47)	-9.63 (21)	<0.001*	2009, 2014 (<0.001/<0.001)
High body-mass index	33.73	29.02	-0.66 (-0.82 to -0.50)	-8.60 (21)	<0.001*	2009, 2013 (<0.001/0.007)
Diet low in whole grains	23.53	21.37	-0.48 (-0.61 to -0.34)	-7.35 (21)	<0.001*	2010, 2014 (<0.001/0.049)
Diet low in fruits	28.27	17.61	-1.99 (-2.15 to -1.82)	-25.24 (22)	<0.001*	2020 (0.007/0.160)
Diet low in vegetables	24.25	17.36	-1.51 (-1.65 to -1.37)	-22.83 (21)	<0.001*	2010, 2014 (0.005/0.028)
Smoking	4.45	5.09	+0.64 (+0.47 to +0.82)	7.71 (21)	<0.001*	2009, 2014 (<0.001/0.018)
Secondhand smoke	14.49	15.89	+0.42 (+0.29 to +0.55)	6.63 (21)	<0.001*	2010, 2014 (<0.001/0.033)
Diet high in sodium	7.33	7.07	-0.07 (-0.19 to +0.06)	-1.13 (21)	0.273	2009, 2013 (<0.001/<0.001)
Low physical activity	14.94	12.19	-0.83 (-0.95 to -0.70)	-13.64 (21)	<0.001*	2009, 2013 (<0.001/<0.001)
Diet high in processed meat	0.51	0.44	-0.55 (-0.77 to -0.33)	-5.13 (21)	<0.001*	2003, 2015 (<0.001/<0.001)
Diet high in sugar-sweetened beverages	0.07	0.10	+1.41 (+1.02 to +1.80)	7.54 (21)	<0.001*	2003, 2014 (<0.001/<0.001)
All risk factors	313.80	243.86	-1.03 (-1.16 to -0.90)	-16.41 (21)	<0.001*	2009, 2013 (<0.001/<0.001)

**Figure 1:**
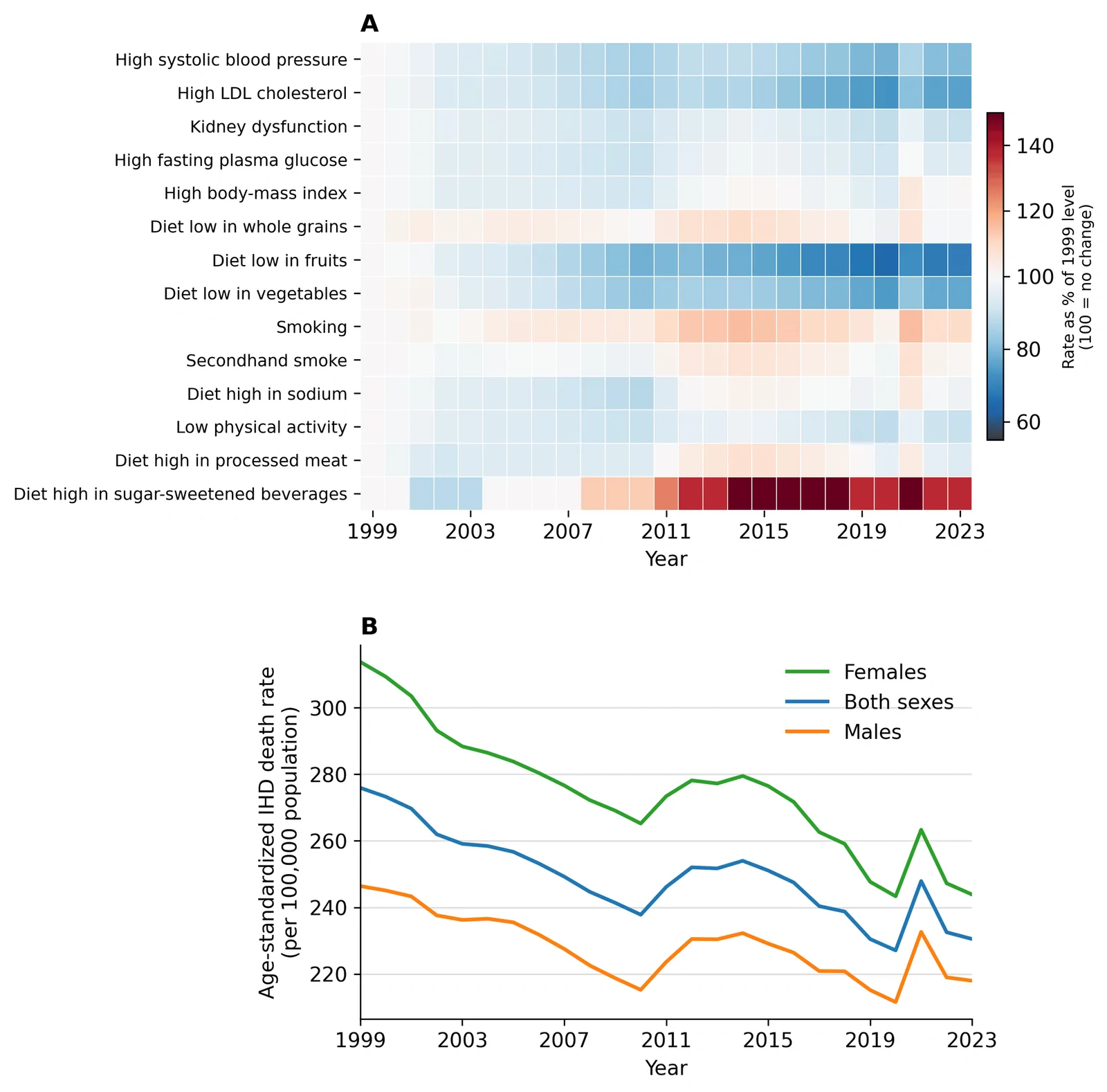
Trends in age-standardized ischemic heart disease death rate attributable to the 14 selected risk factors (A) and to the GBD composite of all risk factors (B) (Afghanistan, 1999-2023). (A) Heat map of the age-standardized IHD death rate for the 14 individual selected risk factors (both sexes), indexed to each risk factor's own 1999 value (= 100). Cell color gives the rate in each year as a percentage of that risk factor's 1999 rate: blue cells are below the 1999 level and red cells above it, independent of the risk factor's absolute magnitude. Risk factors are ordered from top to bottom by their 2023 age-standardized rate. The absolute rates and trend statistics (AAPC, 95% CI, p-value) for every risk factor and sex stratum are in Table 3. (B) Composite age-standardized IHD death rate attributable to all risk factors combined (GBD composite, which also includes risk factors outside the 14), by sex, 1999-2023; the y-axis is the rate per 100,000 population (point estimate). IHD: ischemic heart disease; AAPC: average annual percent change; CI: confidence interval; GBD: Global Burden of Disease Figure developed by the authors from GBD 2023 data obtained through the GBD Results Tool [13].

Table 3 reports the trend statistics from the segmented log-linear analysis for every risk factor, stratified by sex (see Methods). These are exploratory estimates from our own approximation to the NCI Joinpoint approach, and the AAPC confidence intervals and p-values are conditional on the selected joinpoints. A segmented trend (at least one statistically significant joinpoint) was selected in 41 of the 45 risk factor-by-sex series tested; only high LDL cholesterol (all three sex strata) and diet low in fruits (males) were best described by a single straight-line trend across the full 25 years. Where a joinpoint was detected, it was usually located in 2009-2013 (most often 2009 and 2013), with a small number of series bending earlier (2002, 2003) or later (2020), across otherwise unrelated risk factors and both sexes, a pattern discussed further in the Discussion.

A single straight line misstates the rate of change on either side of a bend, so several AAPC estimates changed once joinpoints were allowed, and in most cases, the decline became steeper than the single-line model had shown. This was the case for high systolic blood pressure (AAPC: −0.95%, 95% CI: −1.09 to −0.81; p < 0.001; joinpoints 2009 and 2013) and kidney dysfunction (AAPC: −0.45%; p < 0.001; joinpoints 2009 and 2013). High fasting plasma glucose, which the single-line model had called non-significant (p = 0.420), showed a significant decline once the 2009-2013 segment was accounted for (AAPC: −0.26%; p<0.001). High LDL cholesterol had no joinpoint and kept its single-line estimate (AAPC: −1.14%; p < 0.001). Diet low in fruits (AAPC: −1.55%; p < 0.001) and diet low in vegetables (AAPC: −1.21%; p < 0.001) also continued to decline significantly.

Only two risk factors still showed a significant rise after joinpoints were allowed: diet high in sugar-sweetened beverages (AAPC: +1.28%, 95% CI: +0.94 to +1.63; p < 0.001; joinpoints 2002 and 2015) and smoking (AAPC: +0.37%; p < 0.001; joinpoint 2014). Both estimates are smaller than the single-line model gave (+2.40% and +0.45%) but remain significant in the segmented model.

In the segmented model, three risk factors that had been identified as increasing in the single-line model ceased to be significant: a diet high in processed meat (revised AAPC: −0.07%; p = 0.339), a diet high in sodium (AAPC: −0.05%; p = 0.411) and exposure to secondhand smoke (AAPC: +0.04%; p = 0.586). These factors do not differ from zero when the segmented trend and its permutation test are taken into account. Similarly, a high body-mass index (AAPC: −0.03%; p = 0.715) and a diet low in whole grains (AAPC: −0.10%; p = 0.166) also remained non-significant, agreeing with the results from the single-line model.

Because many series were tested, we checked the AAPC results against a stricter threshold. Of the 45 AAPC tests in Table 3, 34 were significant at 0.05, and all 34 had p < 0.001, which is below the Bonferroni threshold of 0.0011. These AAPC results therefore do not depend on the lack of adjustment. The same cannot be said for the permutation tests that selected the joinpoints, several of which had p-values between 0.001 and 0.05, or for some results in the comparator countries (for example, Pakistan, p = 0.005 to 0.045). Those findings should be read with more caution.

As shown in Table 3 and Figure 2, in 2023, the composite rate was higher among females (243.86 per 100,000) than among males (217.95). This difference was primarily due to metabolic risk factors, since high systolic blood pressure, high LDL cholesterol, high fasting plasma glucose, kidney dysfunction, and a high body-mass index were all higher in females than in males throughout the study period. In contrast, smoking was higher among males (25.83 per 100,000 compared with 5.09 per 100,000 in 2023).

**Figure 2:**
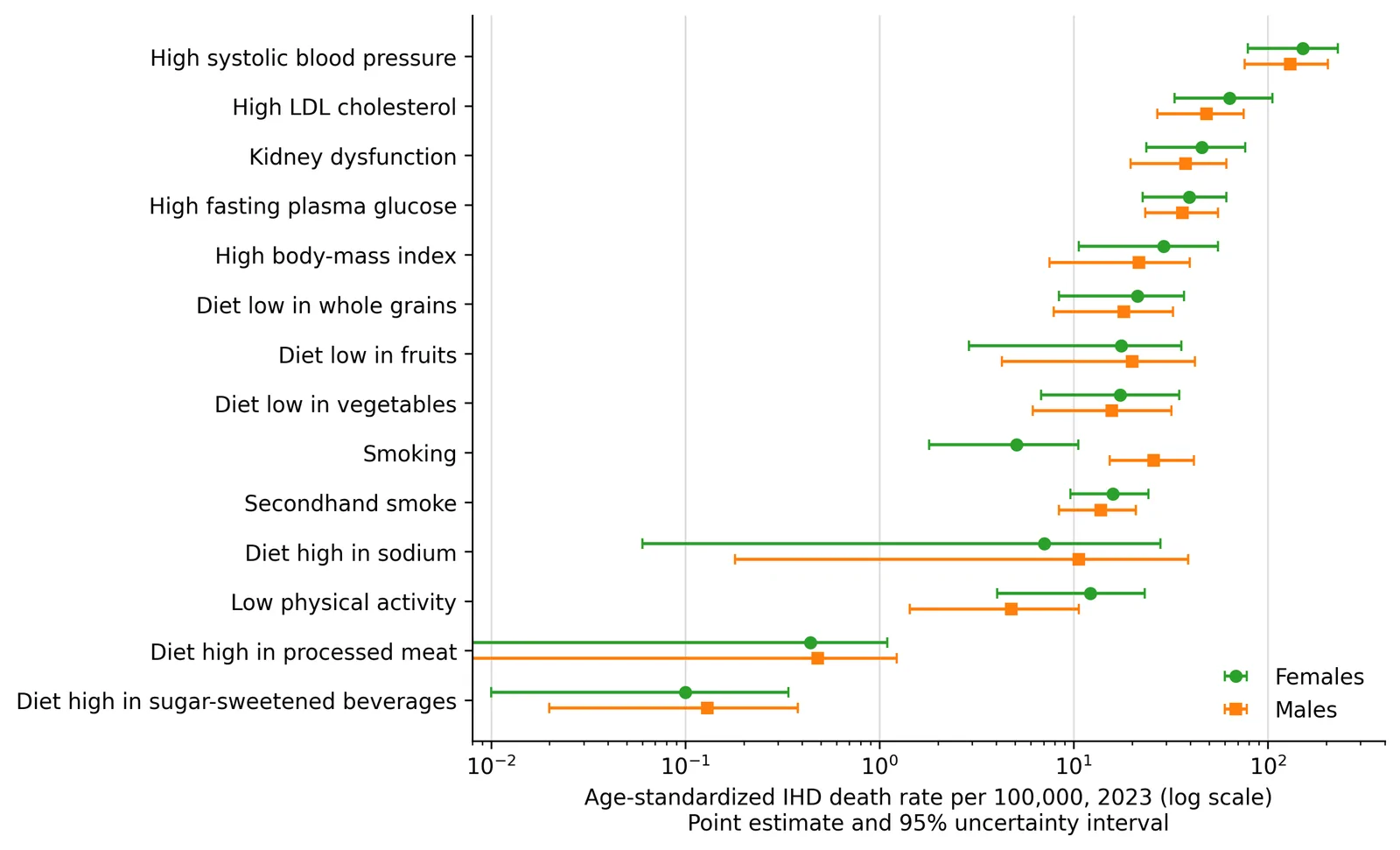
Sex-specific age-standardized ischemic heart disease death rate attributable to each modifiable risk factor, with 95% uncertainty intervals (Afghanistan, 2023). Points are GBD 2023 point estimates of the age-standardized ischemic heart disease (IHD) death rate per 100,000 population in 2023 for females (circles) and males (squares); horizontal bars are 95% uncertainty intervals. The X-axis is logarithmic. Risk factors are ordered by the both-sex 2023 rate. For diet high in processed meat, the lower uncertainty bound is below zero and is truncated at the axis limit. GBD: Global Burden of Disease; LDL: low-density lipoprotein Figure developed by the authors from GBD 2023 data obtained through the GBD Results Tool [13].

We also ran a multiple linear regression (N = 700 observations) to compare the average rates by risk factor, sex, and year. This model is only descriptive, as shown by the results in Table 4.

**Table 4:** Multiple linear regression of age-standardized ischemic heart disease death rate on risk factor category, sex, and calendar year (Afghanistan, 1999-2023). Ordinary least squares regression of the age-standardized ischemic heart disease death rate (per 100,000) on risk factor category, sex, and calendar year (N = 700 observations: 14 risk factors × 25 years × 2 sexes). The model assumes one common linear yearly change for all risk factors and both sexes (no interaction terms). It is a descriptive comparison of average rates and does not describe the trend of individual risk factors (see Table 3). Reference categories: smoking (risk factor) and male (sex). Calendar year was centered at its mean (2011), so the intercept is the predicted rate for smoking among males in 2011. Each coefficient was tested with a two-sided t-test (t = B/SE; 684 degrees of freedom); overall model fit was tested with the F-test; multicollinearity was assessed with the variance inflation factor and serial correlation with the Durbin-Watson statistic (0.03). B: unstandardized regression coefficient; SE: standard error; CI: confidence interval; t: t-statistic; VIF: variance inflation factor (not defined for the intercept); LDL: low-density lipoprotein; model fit: R² = 0.963; adjusted R² = 0.962; F(15, 684) = 1195.1 (p < 0.001); * p ≤ 0.05. Table developed by the authors from GBD 2023 data obtained through the GBD Results Tool [13].

Predictor	B (unstd.)	SE	95% CI	t	p-value	VIF
Intercept (risk=smoking, sex=males, mean year)	11.731	1.116	+9.540 to +13.923	10.51	<0.001*	-
Year (centered, per 1-year increase)	-0.207	0.04	-0.285 to -0.128	-5.17	<0.001*	1
Risk: Diet high in processed meat (ref: smoking)	-14.374	1.525	-17.368 to -11.380	-9.43	<0.001*	1.86
Risk: Diet high in sodium (ref: smoking)	-6.112	1.525	-9.106 to -3.117	-4.01	<0.001*	1.86
Risk: Diet high in sugar-sweetened beverages (ref: smoking)	-14.761	1.525	-17.756 to -11.767	-9.68	<0.001*	1.86
Risk: Diet low in fruits (ref: smoking)	7.591	1.525	+4.597 to +10.586	4.98	<0.001*	1.86
Risk: Diet low in vegetables (ref: smoking)	4.03	1.525	+1.036 to +7.024	2.64	0.008*	1.86
Risk: Diet low in whole grains (ref: smoking)	5.934	1.525	+2.940 to +8.928	3.89	<0.001*	1.86
Risk: High LDL cholesterol (ref: smoking)	49.739	1.525	+46.745 to +52.733	32.62	<0.001*	1.86
Risk: High body-mass index (ref: smoking)	9.887	1.525	+6.893 to +12.882	6.48	<0.001*	1.86
Risk: High fasting plasma glucose (ref: smoking)	23.768	1.525	+20.774 to +26.762	15.59	<0.001*	1.86
Risk: High systolic blood pressure (ref: smoking)	140.919	1.525	+137.925 to +143.913	92.41	<0.001*	1.86
Risk: Kidney dysfunction (ref: smoking)	29.471	1.525	+26.477 to +32.466	19.33	<0.001*	1.86
Risk: Low physical activity (ref: smoking)	-5.792	1.525	-8.787 to -2.798	-3.80	<0.001*	1.86
Risk: Secondhand smoke (ref: smoking)	0.109	1.525	-2.885 to +3.103	0.07	0.943	1.86
Sex: Female (ref: male)	6.259	0.576	+5.128 to +7.391	10.86	<0.001*	1

Risk factor category, calendar year, and sex were all significantly associated with the average rate (all p < 0.05). Relative to smoking, high systolic blood pressure had by far the largest coefficient (B = +140.92, 95% CI: +137.93 to +143.91; p < 0.001), followed by high LDL cholesterol (B = +49.74; p < 0.001) and kidney dysfunction (B = +29.47; p < 0.001). Diet high in sugar-sweetened beverages (B = −14.76; p < 0.001) and diet high in processed meat (B = −14.37; p < 0.001) had the largest negative coefficients, in line with their low absolute rates across the study period. Sugar-sweetened beverages therefore have a low absolute-rate coefficient even though it is one of only two risk factors with a significant rising trend (Table 3).

Calendar year stayed significant after adjustment for risk factor and sex. On average across the 14 risk factors and both sexes, the rate fell by 0.207 per 100,000 each year (95% CI: -0.285 to -0.128; p < 0.001). This is a pooled average. It does not mean that every risk factor declined: the model has no year-by-risk-factor or year-by-sex terms, and Table 3 shows that some risk factors fell, some did not change, and some rose. Rates in women were 6.26 per 100,000 higher than in men (95% CI: +5.13 to +7.39; p < 0.001).

The average difference was smaller than the unadjusted gap of 25.91 per 100,000 in the composite rate (Table 3), because the regression coefficient reflects the average sex difference across all 14 risk-factor categories, each given equal weight. In contrast, the composite rate is mainly influenced by the risk factors with the largest effect, namely, high systolic blood pressure and high LDL cholesterol, for which the excess in females is greatest.

Secondhand smoke did not differ from smoking, the reference category (B = +0.11; p = 0.943). The model explained 96.3% of the variance (R² = 0.963, adjusted R² = 0.962; F(15, 684) = 1195.1; p < 0.001). The Durbin-Watson statistic within each risk factor-by-sex series was 0.03. We expected a low value because each series is a run of consecutive years.

Regional comparison (Table 5 and Figure 3): Afghanistan's composite age-standardized IHD death rate attributable to all risk factors (230.49 per 100,000 in 2023) was the second-highest among the four countries, below Tajikistan (316.52), and almost twice the rates in Pakistan (124.72) and Iran (117.29). The four countries followed different paths from 1999 to 2023. Iran's rate fell sharply, from 222.48 to 117.29 (AAPC: −2.82%, 95% CI: −2.94 to −2.69; p < 0.001). Afghanistan also declined, though more slowly (AAPC: −0.77%). Pakistan barely moved, rising from 120.15 to 124.72 (AAPC: +0.09%, 95% CI: −0.15 to +0.34; p = 0.444), which was not significant. Tajikistan went the other way, rising from 268.32 to 316.52 (AAPC: +0.78%, 95% CI: +0.55 to +1.00; p < 0.001).

**Table 5:** Age-standardized ischemic heart disease death rates in Afghanistan, Pakistan, Iran, and Tajikistan in 1999 and 2023, for all risk factors together (GBD composite) and for the main individual risk factors. The average annual percent change (AAPC) is from our segmented trend analysis and is exploratory. Rates are age-standardized IHD deaths per 100,000, both sexes. % change is the relative change in the rate from 1999 to 2023. % of IHD deaths is the share of all IHD deaths (all ages, both sexes) attributable to the risk factor in 2023. Joinpoints were chosen by the permutation procedure described in the Methods (up to two joinpoints; 4,499 permutations); AAPC is the segment-length-weighted average annual percent change with delta-method 95% CI; * p ≤ 0.05. "All risk factors" is the jointly modeled GBD composite, which includes GBD risk factors outside the 14 selected for this study. The CI and p-value of each AAPC are conditional on the selected joinpoints, and p-values are not adjusted for multiple comparisons. Iran = Iran (Islamic Republic of). t (df): t statistic and degrees of freedom for the test that the AAPC differs from zero; values in parentheses after the joinpoint years are the permutation-test p-values (0 versus ≥1 joinpoint and, where applicable, 1 versus 2 joinpoints). Table developed by the authors from GBD 2023 data obtained through the GBD Results Tool [13].

Country/Risk Factor	1999 Rate	2023 Rate	% Change	% of IHD Deaths, 2023	Joinpoint Year(s) (Permutation p)	AAPC, % (95% CI)	t (df)	p-value
AFGHANISTAN								
All risk factors	275.94	230.49	-16.5	93.88	2009, 2013 (0.003/0.003)	-0.77 (-0.90 to -0.63)	-11.95 (21)	<0.001*
High systolic blood pressure	174.93	140.15	-19.9	55.17	2009, 2013 (0.007/0.010)	-0.95 (-1.09 to -0.81)	-14.05 (21)	<0.001*
High LDL cholesterol	74.11	55.99	-24.5	27.12	None (linear) (0.120)	-1.14 (-1.30 to -0.98)	-14.82 (23)	<0.001*
High fasting plasma glucose	40.44	37.94	-6.2	14.67	2009, 2013 (<0.001/0.002)	-0.26 (-0.39 to -0.13)	-4.18 (21)	<0.001*
Kidney dysfunction	46.80	41.77	-10.7	15.32	2009, 2013 (0.001/<0.001)	-0.45 (-0.57 to -0.33)	-7.78 (21)	<0.001*
High body-mass index	25.42	25.58	+0.6	12.85	2009, 2013 (0.001/0.011)	-0.03 (-0.19 to +0.13)	-0.37 (21)	0.715
Diet high in sugar-sweetened beverages	0.08	0.11	+37.5	0.07	2002, 2015 (<0.001/<0.001)	+1.28 (+0.94 to +1.63)	7.71 (21)	<0.001*
Smoking	13.53	14.78	+9.2	8.44	2014 (0.002/0.140)	+0.37 (+0.22 to +0.53)	4.96 (22)	<0.001*
PAKISTAN								
All risk factors	120.15	124.72	+3.8	95.50	2016, 2020 (0.005/0.001)	+0.09 (-0.15 to +0.34)	0.78 (21)	0.444
High systolic blood pressure	64.20	68.03	+6.0	50.50	2016, 2020 (0.001/<0.001)	+0.24 (+0.01 to +0.47)	2.14 (21)	0.045*
High LDL cholesterol	37.50	40.98	+9.3	34.15	2016, 2020 (0.011/0.003)	+0.30 (+0.05 to +0.56)	2.47 (21)	0.022*
High fasting plasma glucose	17.93	19.52	+8.9	14.45	2016, 2020 (<0.001/<0.001)	+0.26 (+0.03 to +0.49)	2.37 (21)	0.027*
Kidney dysfunction	21.61	23.67	+9.5	16.60	2016, 2020 (<0.001/<0.001)	+0.34 (+0.12 to +0.57)	3.17 (21)	0.005*
High body-mass index	6.44	12.36	+91.9	10.89	2015, 2020 (0.003/0.009)	+2.66 (+2.38 to +2.93)	20.23 (21)	<0.001*
Diet high in sugar-sweetened beverages	0.12	0.15	+25.0	0.14	2010 (0.013/0.170)	+0.62 (+0.39 to +0.85)	5.54 (22)	<0.001*
Smoking	26.87	20.52	-23.6	16.57	2014, 2020 (<0.001/0.001)	-1.35 (-1.60 to -1.10)	-11.19 (21)	<0.001*
IRAN								
All risk factors	222.48	117.29	-47.3	92.80	2010, 2017 (<0.001/0.007)	-2.82 (-2.94 to -2.69)	-45.72 (21)	<0.001*
High systolic blood pressure	125.42	69.58	-44.5	54.19	2009, 2018 (<0.001/0.018)	-2.62 (-2.76 to -2.49)	-39.48 (21)	<0.001*
High LDL cholesterol	77.72	37.94	-51.2	32.90	2012, 2017 (<0.001/0.012)	-3.18 (-3.31 to -3.05)	-50.29 (21)	<0.001*
High fasting plasma glucose	18.20	13.59	-25.3	10.37	2009, 2017 (<0.001/0.018)	-1.40 (-1.54 to -1.27)	-21.29 (21)	<0.001*
Kidney dysfunction	48.84	28.80	-41.0	21.21	2002, 2006 (<0.001/0.044)	-2.31 (-2.44 to -2.17)	-35.89 (21)	<0.001*
High body-mass index	26.16	20.75	-20.7	18.21	2010, 2017 (<0.001/0.002)	-1.16 (-1.30 to -1.02)	-17.44 (21)	<0.001*
Diet high in sugar-sweetened beverages	1.00	0.56	-44.0	0.56	2007 (0.001/0.197)	-2.47 (-2.59 to -2.35)	-42.50 (22)	<0.001*
Smoking	24.66	12.36	-49.9	12.89	2003, 2009 (0.001/0.013)	-2.85 (-3.00 to -2.71)	-41.20 (21)	<0.001*
TAJIKISTAN								
All risk factors	268.32	316.52	+18.0	95.19	2003, 2020 (<0.001/0.001)	+0.78 (+0.55 to +1.00)	7.24 (21)	<0.001*
High systolic blood pressure	172.57	224.29	+30.0	66.00	2003, 2020 (<0.001/<0.001)	+1.19 (+0.97 to +1.41)	11.44 (21)	<0.001*
High LDL cholesterol	69.70	74.82	+7.3	25.83	2003, 2020 (<0.001/0.045)	+0.40 (+0.15 to +0.66)	3.30 (21)	0.003*
High fasting plasma glucose	32.36	48.58	+50.1	13.90	2003, 2020 (<0.001/<0.001)	+1.80 (+1.59 to +2.01)	17.96 (21)	<0.001*
Kidney dysfunction	51.16	62.11	+21.4	17.19	2003, 2020 (<0.001/0.002)	+0.90 (+0.69 to +1.11)	8.85 (21)	<0.001*
High body-mass index	34.06	44.12	+29.5	15.62	2002, 2020 (<0.001/0.043)	+1.20 (+0.91 to +1.49)	8.54 (21)	<0.001*
Diet high in sugar-sweetened beverages	0.12	0.21	+75.0	0.08	2007, 2014 (<0.001/<0.001)	+2.27 (+2.05 to +2.50)	21.05 (21)	<0.001*
Smoking	25.48	20.33	-20.2	8.49	2020 (<0.001/0.606)	-1.00 (-1.31 to -0.69)	-6.75 (22)	<0.001*

**Figure 3:**
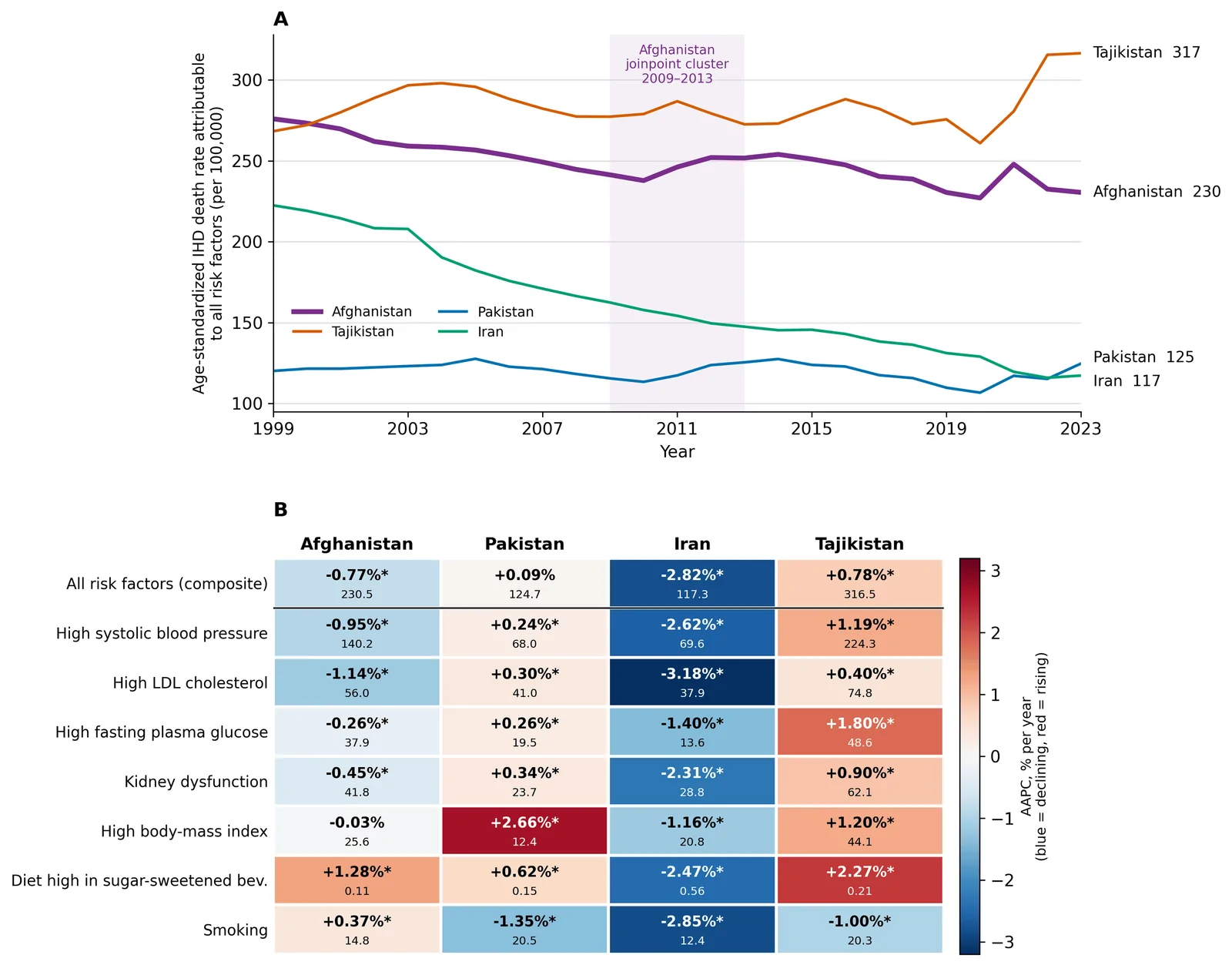
Regional comparison of ischemic heart disease mortality attributable to the GBD composite of all risk factors and to seven selected risk factors, Afghanistan versus Pakistan, Iran, and Tajikistan (1999-2023). (A) Composite age-standardized IHD death rate attributable to all risk factors in Afghanistan, Pakistan, Iran, and Tajikistan, 1999-2023 (both sexes, per 100,000 population). Afghanistan is drawn with a heavier line, the shaded band marks the 2009-2013 period in which most of Afghanistan's joinpoints were detected (Table 3), and the end labels give the 2023 rate. (B) Heat map of the segmented-model average annual percent change (AAPC, 1999-2023) for the composite category and seven individual risk factors. Cell color shows the AAPC (blue = declining, red = rising), the bold number is the AAPC (* p ≤ 0.05), and the small number is the 2023 age-standardized rate per 100,000 population. IHD: ischemic heart disease; AAPC: average annual percent change; LDL: low-density lipoprotein Figure developed by the authors from GBD 2023 data obtained through the GBD Results Tool [13].

In all four countries, high systolic blood pressure came first and high LDL cholesterol second. In 2023, blood pressure accounted for 55.2% of IHD deaths in Afghanistan, 50.5% in Pakistan, 54.2% in Iran, and 66.0% in Tajikistan. The shares for LDL cholesterol were 27.1%, 34.2%, 32.9%, and 25.8%. The direction of change was not the same everywhere. Rates for both risk factors fell in Afghanistan and Iran. In Pakistan, both rose (AAPC: +0.24% for blood pressure and AAPC: +0.30% for LDL cholesterol), and in Tajikistan, they rose faster (AAPC: +1.19% and AAPC: +0.40%).

Two risk factors separated Afghanistan from its neighbors. Smoking-attributable IHD mortality rose in Afghanistan (AAPC: +0.37%) but fell in all three neighbors (Pakistan: −1.35%, Iran: −2.85%, Tajikistan: −1.00%; all p < 0.001). The high body-mass index rate was flat in Afghanistan (AAPC: −0.03%; p = 0.715) but rose steeply in Pakistan (AAPC: +2.66%, 95% CI: +2.38 to +2.93) and Tajikistan (+1.20%), and fell in Iran (AAPC: −1.16%). Sugar-sweetened beverage-attributable rates rose in Afghanistan, Pakistan (AAPC: +0.62%), and Tajikistan (AAPC: +2.27%) and fell in Iran (AAPC: −2.47%).

Some features of the Afghanistan series were shared by its neighbors (Figure 3A). The dip in 2010 and rebound to 2014 that produced the 2009 and 2013 joinpoints in Afghanistan (237.8 to 254.0 per 100,000, +6.8%) appeared almost exactly in Pakistan (113.4 to 127.5, +12.4%), while Tajikistan showed only a brief rise in 2011 (278.9 to 286.8) and Iran declined smoothly through these years. The one-year rise in 2021 in Afghanistan (+9.2% on 2020) was matched in Pakistan (+9.8%) and Tajikistan (+7.5%) but not in Iran (−7.3%); Pakistan and Tajikistan each had a joinpoint in 2020.

## Discussion

Over the course of the GBD 2023 estimates spanning 25 years, the age-standardized death rate from IHD in Afghanistan attributable to the GBD composite of all risk factors decreased by 16.5% (with the segmented-model AAPC at −0.77% per year), whereas the total number of deaths continued to increase. This pattern is consistent with population growth and aging, although we did not decompose it formally. The two main metabolic risk factors, namely, high systolic blood pressure and high LDL cholesterol, both showed a decline. However, a smaller number of dietary and behavioral risk factors increased, even though fewer than the single-line model had initially indicated.

The pattern we observe for Afghanistan in 2023 is in line with the global GBD 2021 analysis of IHD, which likewise identified high systolic blood pressure and high LDL cholesterol as the two main risk factors on a global level (they accounted for 52.2% and 30.1% of global IHD deaths, respectively) and observed a 31.6% decrease in the global age-standardized IHD death rate between 1990 and 2021 [2]. The order of risk factors that we found for Afghanistan in 2023 (high systolic blood pressure being responsible for 55.17% and high LDL cholesterol for 27.12% of the attributable deaths) is very similar to this global pattern.

Nevertheless, the rate of decline in Afghanistan from 1999 to 2023 (AAPC: −0.77% per year, composite) is slower than the global trend seen between 1990 and 2021, even when the different and non-overlapping time periods are taken into account; this is consistent with the finding from Afghanistan's 2018 STEPS survey [8] of a persistently high prevalence of hypertension (25% of adults), and also with the descriptions of a health system that has struggled to maintain chronic disease detection and control services during repeated periods of conflict and, more recently, because of a decrease in funding [10,11].

Compared with its neighbors, in 2023 Afghanistan's overall rate was nearly twice that of Pakistan and Iran and was only exceeded by Tajikistan. Moreover, its decline of 0.77% per year was less than one-third of Iran's 2.82%. In Iran, the decrease was due to falling mortality attributable to blood pressure, LDL, smoking, and body mass index, which is consistent with a long-standing primary care program for non-communicable diseases and a relatively complete system of death registration [12,26]. In contrast, Pakistan's trend remained level, and that of Tajikistan increased: in both countries the rates attributable to blood pressure and LDL were on the rise, and in Pakistan mortality attributable to body mass index was increasing rapidly (by +2.66% per year) and in Tajikistan it was rising as well (by +1.20%), whereas in Afghanistan it had stayed the same.

Two factors distinguish Afghanistan. There, mortality attributable to smoking was increasing, while in all three neighboring countries, it was decreasing, which means that tobacco control should be a particular priority in Afghanistan. The lack of an increasing body mass index signal might be due to lower levels of adiposity or simply to sparse exposure data, and this point needs to be confirmed by primary surveys.

With joinpoints in the model, only two dietary or behavioral risk factors still showed a significant rise. The rate attributable to sugar-sweetened beverages rose faster than that of any other risk factor (AAPC: +1.28% per year). Cohort meta-analyses have linked higher intake of these drinks to coronary heart disease in a dose-response manner [27]. The rate attributable to smoking also rose (AAPC: +0.37% per year). In 2023, it was about five times higher in men than in women (25.83 versus 5.09 per 100,000). This fits Afghan tobacco surveys, where few people smoke overall, and most of those who do are men [8].

Processed meat and sodium, which the single-line model had identified, were not significant in the segmented model (with both p-values greater than 0.3) and should not be interpreted as increasing. The two significant increases are consistent with the early stage of the nutritional and behavioral transition that has occurred in low- and middle-income countries [28], occurring in a population that already bears the burden of hypertension and dysglycemia [8,9]. Although both risk factors are still small in comparison to blood pressure and LDL cholesterol, they are the ones trending in the wrong direction and therefore warrant their own prevention strategies.

Women show higher levels than men in most metabolically related risk factors (such as high systolic blood pressure, high LDL cholesterol, high fasting plasma glucose, kidney dysfunction, and a high body-mass index), which contrasts with the male predominance usually seen in the overall incidence of IHD in many populations, even as sex-specific patterning of individual cardiovascular risk factors is increasingly recognized [29]; this pattern should be confirmed using primary epidemiological data from Afghanistan.

Our multiple regression model helps us better understand the magnitude of this difference. After taking into account the risk factor category and the calendar year, the average female excess across all 14 risk factor categories was much smaller (B = +6.26 per 100,000) than the unadjusted difference in the combined rate (25.91 per 100,000), showing that the overall female excess is concentrated in and mainly caused by the small number of metabolic risk factors with the highest magnitude rather than being a consistent feature of all risk factor categories.

One of the findings from the segmented trend analysis has to be interpreted with care. In Afghanistan, the breaks in trend cluster between 2009 and 2013: of the 45 risk factor-by-sex series, 41 had a significant joinpoint, and most of these occurred within or just after this period, regardless of the risk factor or sex. It is improbable that 14 separate exposures should all have changed at the same time.

The neighboring countries help identify the cause. A similar dip in 2010 followed by a rise to 2014 is almost exactly seen in Pakistan; Tajikistan shows a brief rise in 2011, while Iran, whose GBD estimates are based on a much more complete death-registration system [12], showed a smooth decline over these years. The fact that the bend is limited to the countries with sparse data suggests that the change is due to a common input or covariate affecting the GBD models for these areas, for example, a new round of regional surveys or revised population estimates, rather than indicating a real event that took place simultaneously in three countries with different health systems. Since our data cannot distinguish between a modeling artifact and an actual regional shift, we note the inflection in the period 2009-2013 rather than attempt to interpret it.

The composite rate rose in 2021 in both men and women and fell again the following year (Figure 1B). That was the year of Afghanistan's political transition, when health services and foreign aid were disrupted [10,11,30]. However, the rise was not limited to Afghanistan. Both Pakistan (+9.8%) and Tajikistan (+7.5%) showed a similar rise in the same year, and both had a joinpoint in 2020, whereas Iran did not. We did not test the cause of this rise, and our data cannot identify it. One possible explanation is a regional effect of the COVID-19 pandemic period, whether through direct cardiovascular deaths, the interruption of care for chronic diseases, or the way excess deaths in 2020 and 2021 were assigned to causes in the GBD 2023 models [31]. We offer this only as a hypothesis. The 2021 pattern could equally come from uncertainty in the modeled estimates, from changes in the data inputs for these countries, or from other regional factors that we did not measure. Disruptions to health services in Afghanistan may also have played a part. These explanations can be separated only when primary surveillance data for those years become available.

Limitations

First, the GBD estimates for Afghanistan are based on modeling rather than on data from a complete vital registration and cause-of-death system and are thus subject to greater uncertainty than in countries with more developed health information systems; this higher level of uncertainty is shown by the wide 95% uncertainty intervals given for several risk factors, especially the smaller dietary risks (Figure 2). Our trend analysis used the GBD point estimates only, so the confidence intervals of the AAPC do not include this uncertainty. For every risk factor, the 95% uncertainty intervals of the 1999 and 2023 rates overlap, so the trends reported here are trends in the GBD point estimates.

Second, the analysis was limited to 1999-2023; trends during 1990-1998, which the GBD series also covers, were not examined.

Third, the various individual risk factor categories are not mutually exclusive and operate via overlapping and mediated biological pathways (for example, a high body-mass index leads to both high systolic blood pressure [32] and high fasting plasma glucose [33]), so the percentages in Table 2 and the regression coefficients in Table 4 should not be added together or interpreted as completely independent and additive contributions. The regression also assumes one common linear yearly change for all risk factors and both sexes, with no interaction terms. It is a descriptive comparison of average rates and does not represent the time pattern of each risk factor, which Table 3 shows to be different between risk factors and often not linear. The regression residuals are also strongly autocorrelated (Durbin-Watson 0.03), so its standard errors and p-values are too small and should not be used for formal inference.

Fourth, this is an ecological analysis at the aggregate population level using summary statistics; therefore, it cannot be used to make any individual-level causal inferences.

Fifth, the segmented trend analysis presented in Table 3 is our own version of the permutation-based model-selection method employed by the NCI Joinpoint Regression Program, not a direct run of that software; it applies a sequential two-step test (first comparing 0 with the best of one or two joinpoints, then comparing one with two) at a nominal α level of 0.05 for each step rather than using the NCI's exact multiple-comparison-corrected search procedure. The joinpoint positions and the AAPCs are therefore exploratory estimates from an approximation, and they are not equivalent to output of the NCI program. In addition, the confidence intervals and p-values of the AAPC are conditional on the selected model. They do not include the uncertainty from choosing the number and position of the joinpoints from the data, so the real uncertainty is larger than the intervals suggest. These estimates need to be checked with the NCI software itself before they are treated as confirmed.

Sixth, we chose the three comparator countries for geographic and cultural proximity, not matched on data quality. Iran's GBD estimates rest on far more complete death-registration inputs than those of Afghanistan, Pakistan, or Tajikistan, so differences in uncertainty, as well as in true burden, contribute to the contrasts in Table 5, and the comparison was limited to both-sex, age-standardized death rates without sex stratification or regression.

Seventh, we carried out many significance tests across risk factors, sexes, and countries and did not adjust the p-values for multiple comparisons. All 34 significant AAPCs in Table 3 had p < 0.001 and would also pass a Bonferroni threshold (0.05/45 = 0.0011), but several of the joinpoint permutation tests and some results for the comparator countries would not. Some of these findings may be false positives, and the individual trend analyses should be treated as exploratory.

Eighth, we did not decompose the change in the number of deaths into population growth, population aging, and changes in age-specific rates, and we did not test any explanation for the rise in 2021. What we say on these two points is an interpretation that fits the data. It is not the result of a formal analysis.

Ninth, the 14 risk factors were chosen to cover behavioral, dietary, and metabolic exposures. Environmental exposures, such as air pollution, lead, and non-optimal temperature, can also be modified, mainly through policy. They are part of the GBD composite but were not analyzed one by one in this study.

## Conclusions

From 1999 to 2023, the age-standardized burden of mortality due to ischemic heart disease in Afghanistan that can be attributed to all GBD risk factors combined decreased. Among the 14 selected behavioral, dietary, and metabolic risk factors, the rates attributable to high systolic blood pressure and high LDL cholesterol both fell, but these two factors remain by far the biggest contributors to deaths from IHD. Our exploratory segmented log-linear trend analysis, which approximates the NCI Joinpoint method but is not the same as it, suggested that the decline was more rapid than a simple linear trend indicates, with a common point of inflection around 2009-2013 for most risk factors, which may reflect a change in the underlying GBD modeling inputs for Afghanistan rather than a specific real-world event.

At the same time, rates linked to the consumption of sugar-sweetened beverages and smoking increased. Both increases remained significant in the segmented analysis, whereas the apparent rise in processed meat and sodium intake did not. These trend results are conditional on the selected model and were not adjusted for multiple comparisons, so they should be confirmed in further work. The higher levels found in women for most metabolic risk factors warrant further investigation. The results show that it is necessary to continue to give priority, at the population level, to the control of hypertension and lipid levels in Afghanistan, together with the development of specific prevention programs aimed at sugar-sweetened beverage consumption and smoking. Compared with its neighbors, Afghanistan's burden remains among the highest in the region, and its progress lags well behind Iran's, but the direction of change is better than in Pakistan or Tajikistan; the rising smoking-attributable mortality that separates Afghanistan from all three neighbors is the clearest regionally specific target for prevention. Finally, Afghanistan's primary vital registration and non-communicable disease surveillance capabilities need strengthening to reduce current modeling uncertainty in the country's GBD estimates.
